# Gynæcological Society of Boston

**Published:** 1873-01

**Authors:** 


GYNÆCOLOGICAL SOCIETY OF BOSTON.—The Senior Editor of this journal acknowledges and highly appreciates the compliment bestowed in his election as a corresponding member of the Gynæcological Society of Boston. Entirely unsolicited and unexpected, it is taken as an evidence of the existence in that body of a fraternal feeling which, among those really devoted to medical science, knows no North, South, East or West, but recognizes as brethren all who are devoting their lives to the relief of suffering humanity.

